# Ultraviolet imager on Venus orbiter *Akatsuki* and its initial results

**DOI:** 10.1186/s40623-017-0772-6

**Published:** 2018-02-12

**Authors:** Atsushi Yamazaki, Manabu Yamada, Yeon Joo Lee, Shigeto Watanabe, Takeshi Horinouchi, Shin-ya Murakami, Toru Kouyama, Kazunori Ogohara, Takeshi Imamura, Takao M. Sato, Yukio Yamamoto, Tetsuya Fukuhara, Hiroki Ando, Ko-ichiro Sugiyama, Seiko Takagi, Hiroki Kashimura, Shoko Ohtsuki, Naru Hirata, George L. Hashimoto, Makoto Suzuki, Chikako Hirose, Munetaka Ueno, Takehiko Satoh, Takumi Abe, Nobuaki Ishii, Masato Nakamura

**Affiliations:** 1 Institute of Space and Astronautical Science (ISAS), Japan Aerospace Exploration Agency (JAXA), 3-1-1 Yoshinodai, Chuo-ku, Sagamihara, Kanagawa 252-5210 Japan; 2 Department of Earth and Planetary Science, Graduate School of Science, The University of Tokyo, 7-3-1 Hongo, Bunkyo-ku, Tokyo, 113-0033 Japan; 3 Planetary Exploration Research Center (PERC), Chiba Institute of Technology, 2-17-1 Tsudanuma, Narashino, Chiba 275-0016 Japan; 4 Present Address: Graduate School of Frontier Sciences, The University of Tokyo, 5-1-5 Kashiwanoha, Kashiwa, Chiba 277-8561 Japan; 5 Hokkaido Information University, 59-2 Nishinopporo, Ebetsu, Hokkaido 069-8585 Japan; 6 Faculty of Environmental Earth Science, Hokkaido University, N10W5, Sapporo, Hokkaido 060-0810 Japan; 7 Artificial Intelligence Research Center, National Institute of Advanced Industrial Science and Technology, 2-3-26 Aomi, Koto-ku, Tokyo, 135-0064 Japan; 8 School of Engineering, University of Shiga Prefecture, 2500 Hassaka-cho, Hikone, Shiga 522-8533 Japan; 9 Graduate School of Frontier Sciences, The University of Tokyo, 5-1-5 Kashiwanoha, Kashiwa, Chiba 277-8561 Japan; 10 Department of Physics, Rikkyo University, 3-34-1 Nishi-Ikebukuro, Toshima-ku, Tokyo, 171-8501 Japan; 11 Faculty of Science, Kyoto Sangyo University, Motoyama, Kamigamo, Kita-ku, Kyoto, Kyoto 603-8555 Japan; 12 Department of Information Engineering, National Institute of Technology, Matsue College, 14-4 Nishi-Ikuma, Matsue, Shimane 690-8518 Japan; 13 Research and Information Center, Tokai University, 4-1-1 Kitakaname, Hiratsuka, Kanagawa 259-1292 Japan; 14 Present Address: Hokkaido University, N10W5, Sapporo, Hokkaido 060-0810 Japan; 15 Department of Planetology/Center for Planetary Science, Kobe University, 7-1-48, Minamimachi, Minatojima Chuo-ku, Kobe, 650-0047 Japan; 16 School of Commerce, Senshu University, 2-1-1 Higashimita, Tama-ku, Kawasaki, Kabagawa 214-8580 Japan; 17 School of Computer Science and Engineering, The University of Aizu, 90 Kami-Iawase, Tsuruga, Ikki-machi, Aizu-Wakamatsu, Fukushima 965-8580 Japan; 18 Department of Earth Science, Okayama University, 3-1-1 Tsushimanaka, Kita, Okayama 700-8530 Japan; 19 Center for Planetary Science (CPS), Graduate School of Science, Kobe University, 7-1-48 Minatojima-minamimachi, Chuo-ku, Kobe, Hyogo 650-0047 Japan; 20 Department of Space and Astronautical Science, School of Physical Sciences, SOKENDAI, 3-1-1 Yoshinodai, Chuo-ku, Sagamihara, Kanagawa 252-5210 Japan

**Keywords:** Venus orbiter *Akatsuki*, Ultraviolet imager (UVI), UVI performance, UV images of Venus at the cloud top altitude, Initial results of cloud tracking

## Abstract

The ultraviolet imager (UVI) has been developed for the *Akatsuki* spacecraft (Venus Climate Orbiter mission). The UVI takes ultraviolet (UV) images of the solar radiation reflected by the Venusian clouds with narrow bandpass filters centered at the 283 and 365 nm wavelengths. There are absorption bands of SO_2_ and unknown absorbers in these wavelength regions. The UV images provide the spatial distribution of SO_2_ and the unknown absorber around cloud top altitudes. The images also allow us to understand the cloud top morphologies and haze properties. Nominal sequential images with 2-h intervals are used to understand the dynamics of the Venusian atmosphere by estimating the wind vectors at the cloud top altitude, as well as the mass transportation of UV absorbers. The UVI is equipped with off-axial catadioptric optics, two bandpass filters, a diffuser installed in a filter wheel moving with a step motor, and a high sensitivity charge-coupled device with UV coating. The UVI images have spatial resolutions ranging from 200 m to 86 km at sub-spacecraft points. The UVI has been kept in good condition during the extended interplanetary cruise by carefully designed operations that have maintained its temperature maintenance and avoided solar radiation damage. The images have signal-to-noise ratios of over 100 after onboard desmear processing.

## Background

Venus has a higher albedo than Earth, and its clouds scatter solar radiation well. Venus’ image is featureless in the visible light region; however, the observed ultraviolet (UV) images have a high contrast of bright and dark features, which reflects the distribution of UV absorbers. One of the UV absorbers is SO_2_, which has an absorption band in the wavelengths 210–320 nm. Another one is an unknown matter that shows the maximum absorption around 400 nm (Esposito et al. 1997; Pollack et al. 1980). Therefore, in contrast to the featureless visible-wavelength Venus images, the UV images present unique cloud morphologies, including the well-known “Yfeature,” which is even observable from ground-based stations (Dollfus 1975). The Venus UV image observations have about 90 years of history starting from the first ground-based observations (Wright 1927; Ross 1928) to the Hubble space telescope (Na and Esposito 1995); several spacecraft observations were performed using flyby opportunities [Mariner 10 (Murray et al. 1974), Galileo (Belton et al. 1991) and Messenger], and using Venus orbiters [Venera (Ksanfomaliti et al. 1978), Pioneer Venus (Travis et al. 1979; Pollack et al. 1979; Stewart et al. 1979), and Venus Express (Markiewicz et al. 2007a, b; Titov et al. 2008, 2012)].

Venus orbiters can provide astonishing details of cloud features that cannot be resolved by ground-based telescopes, such as polar vortexes, equatorial convective cells, and bright polar “hoods” (Rossow et al. 1980; Titov et al. 2008, 2012). As a new generation Venus orbiter, *Akatsuki* (Nakamura et al. 2007, 2011) was successfully inserted into the Venus orbit in December 2015 (Nakamura et al. 2016), and the onboard ultraviolet imager (UVI) continues the Venus UV observations at 283 and 365 nm. These images are used to investigate the distributions of the absorbers, such as SO_2_ and unknown materials in the Venus mesosphere. Other scientific targets are to derive wind vectors using cloud tracking techniques (e.g., Limaye and Suomi 1981; Moissl et al. 2009; Ogohara et al. 2012; Kouyama et al. 2012; Khatuntsev et al. 2013; Hueso et al. 2015; Ikegawa and Horinouchi 2016; Horinouchi et al. 2017a), and to retrieve the vertical distribution of haze from limb observations.

The H_2_SO_4_ clouds (Esposito et al. 1983, 1997) are formed by photochemical reactions of SO_2_ and H_2_O near the cloud top altitude. However, the process of SO_2_ transport in the Venus atmosphere is not yet well understood. SO_2_ is abundant below the clouds, but it is unclear how SO_2_ is transported to the cloud top region (Ignatiev et al. 2009), where the stratification is static and stable. Moreover, the unknown absorbers are responsible for considerable solar heating at the cloud top level (Crisp and Titov 1997; Lee et al. 2015). This heating results in thermal tides, which play an important role in momentum transport, so could contribute to maintain strong zonal winds near the cloud top level (Takagi and Matsuda 2007; Lebonnois et al. 2016).

First cloud motion measurements on Venus were reported from ground-based images by Boyer (1965), Beebe et al. (1973), and from first spacecraft observations from Mariner 10 flyby images by Suomi (1975), Suomi et al. (1976), Sidi (1976), Limaye (1977), Limaye and Suomi (1981). Winds at the Venusian cloud top have been acquired from the cloud morphologies observed by the Orbiter Cloud Photo-polarimeter (OCPP) on board the Pioneer Venus Orbiter (Rossow et al. 1990), Galileo (flyby) by Toigo et al. (1994), the Venus Monitoring Camera (VMC) on board the Venus Express (Markiewicz et al. 2007b) and other spacecraft missions. The flow is almost zonal and westward, i.e., in the same direction as Venus’ rotation. The wind is called “superrotation” because it is sixty times faster than the winds at ground level on Venus. The speed of the zonal westward wind increases with altitude and reaches ~ 100 m/s (mean value) near the cloud top altitude (Khatuntsev et al. 2013; Hueso et al. 2015). Several generation mechanisms of superrotation have been proposed by many authors (Leovy 1973; Gierasch 1975; Rossow and Williams 1979; Hou and Farrell 1987: Gierasch et al. 1997; Takagi and Matsuda 2007), but the mechanism is still unclear because we do not have enough data on the three-dimensional wind distribution. The coupling among waves, cloud processes and global-scale winds is also important to understand the dynamics of the Venusian atmosphere.

The research goals for UVI data are: (1) large-scale (1000–40,000 km) to mesoscale (1–1000 km) cloud morphologies, (2) three-dimensional haze distribution, (3) interactions between the lower and the middle atmosphere, (4) generation, propagation and dissipation of planetary waves and gravity waves, and their interaction with general circulation, (5) generation of superrotation, (6) distribution of unidentified ultraviolet absorbers, (7) distribution of SO_2_ and the photochemical processes related to H_2_SO_4_ formation, and (8) cloud aerosol microphysical properties.

In this study, we describe the characteristics of UVI, calibration process using ground and onboard measured data, and observation performance and strategy. We show example Venus images taken by UVI, and retrieved wind vectors as a product of the UVI data.

## Instrumentation and observation

### Instrumental design

**Fig. 1:**
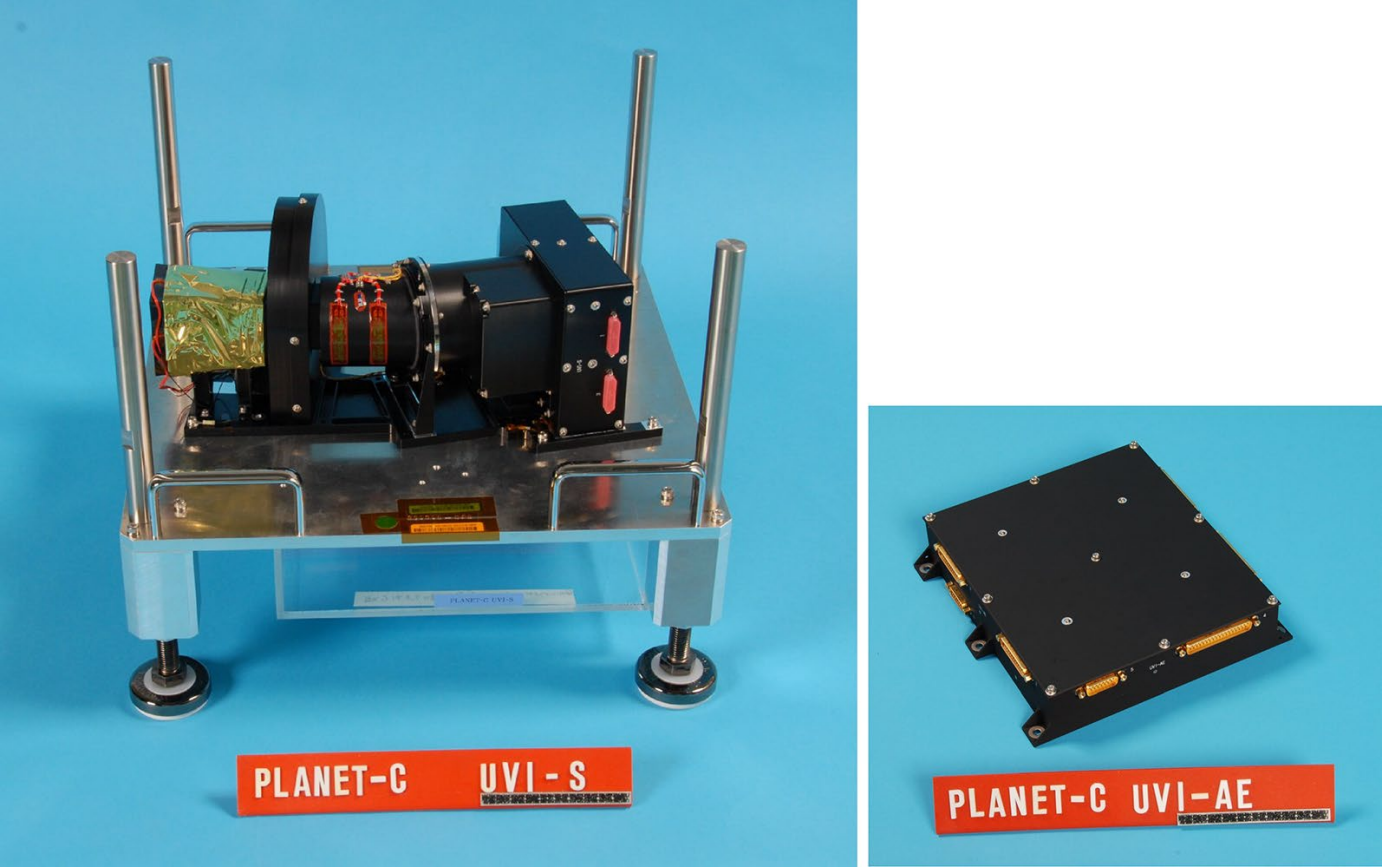
Photographs of UVI-S (left) and UVI-AE (right). The radiator for the CCD cooling is hiding under the baseplate of the instrument in the left panel

**Fig. 2:**
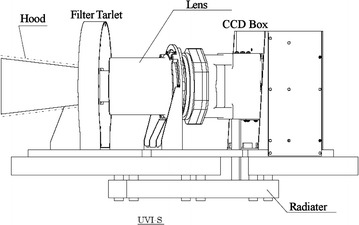
Schematic layout of UVI

The UVI instrument consists of two parts: a sensor (UVI-S) and an analog electronics unit (UVI-AE) (Fig. 1). The UVI-S consists of band pass filters (installed in a filter wheel turret), off-axial catadioptric optics, and a charge-coupled device (CCD) detector with a preamplifier circuit. The instrument layout is shown in Fig. 2. The UVI-AE includes a power supply unit and control and readout circuits of the CCD detector. The electric power consumption is 19 W in the observation mode and 34 W at the filter wheel rotation. The UVI characteristics are shown in Table 1.

**Table 1:** Characteristics of UVI

Observation target	Solar radiation scattered at cloud top
Optics design	Camera with off-axial catadioptric optics
Observational wavelength	283 and 365 nm
Field-of-view	12°
FOV per pixel	0.20 mrads
Spatial resolution	~ 200 m (periapsis)–76 km (60Rv)
*Optics*	
F-number	16
Focal length	63.3 mm
Aperture size	39.89 mm (hood entrance)
Bandpass widths of the filters	14 nm
*Detector*	
CCD	SiCCD (back-illuminated and full-frame transfer)
Pixel number	1024 × 1024 pixels
*CCD control*	
Exposure time	4 ms–11 s
Data depth	12 bit
*Weight (kg)*	
UVI-S	2.9
UVI-AE	1.2
*Size*	
UVI-S	199 mm × 206 mm × 376 mm
UVI-AE	220 mm × 220 mm × 50 mm
*Power (W)*	
Stand-by mode	17
FW movement mode	34
Observation mode	19

The UVI-S has an off-axial catadioptric optics that consists of two lenses and two reflecting surfaces. The optics has a composite focal length of 63.3 mm and an f-number of 16. The 12° × 12° field-of-view (FOV) can capture the whole Venus disk during 97% of one rotation, except over ~ 8 h near a periapsis. The size of the point spread function of the optics is designed to be smaller than 2 pixels of CCD. The filter wheel has four positions. Two interference band pass filters and one diffuser are installed in the filter wheel. The last position is used as a shutter to measure noise level. The interference filters select the observational wavelengths of 283 and 365 nm with a bandpass width of 14 nm. The wavelength of 283 nm is located in the middle of the strong absorption band of SO_2_ (Stewart et al. 1979). The wavelength of 365 nm is in the broad absorption of the unknown UV absorbers, whose strong contrasts enable us to track the cloud morphology easily, as previous Venus orbiter observations had reported. The diffuser is used for the onboard flat field calibration, such as the measurement of the correction of relative sensitivity between pixels. The shutter position is used to obtain the noise counts. The wheel positions are controlled by a step motor and two Hall effect sensors to determine the wheel rotation angle; they are also used to automatically return the wheel to the shutter position.

UVI-S is a full-frame back-illuminated sensor with a UV sensitive coating, a pixel size of 13 μm, and an imaging area of 1024 × 1024 pixels. The angular resolution is 0.012°, which corresponds to spatial resolutions of ~ 200 m and ~ 76 km on the cloud top level in the observations from the altitudes of ~ 1000 km at the periapsis and ~ 60 R_V_ (the radius of Venus) at the apoapsis, respectively. The readout signal of the CCD is 12 bits. The data processing at the Digital Electronics (DE) equipment for the imaging sensors on *Akatsuki*, such as median filtering, subtraction of dark current, and desmearing, is performed after converting the original 12 bits data to 16 bits data. The image is compressed to reduce the size from 2 MB (megabyte) to several hundred KB (kilobyte) using an onboard application program. The compression algorithm is a lossless method and is called “HIREW” (Takada et al. 2007).

### Observations and onboard data processing

The exposure time of UVI can be selected by a single command from 0.004 s to 11 s with 24 steps. The chosen Venus image exposure time is 0.25 s before June 5, 2016 and/or 0.50 s after June 6, 2016 for 283 nm, while the exposure time is 0.046 s for 365 nm from December 7, 2015. The CCD detector with no electrical cooler system is thermally in contact with a cooling radiator on the outside panel of the spacecraft body to reduce the dark current. The radiator has sufficient area to cool the CCD to less than 9 °C in the observation mode. The signal-to-noise ratio of raw images exceeds 10 at this temperature before image data processing.

The CCD has no mechanical shutter; thus smear noise in the transfer from the image area to the storage area on the chip degrades the signal-to-noise ratio of the obtained image, especially in the case of short exposure. In the nominal operation of UVI, 18 images (6 Venus and 12 shutter images) are used for smear correction for each wavelength for the onboard data processing at the DE equipment. A set of three images taken in the same condition yields one median image. Letting *T* be the chosen exposure time, the breakdown of 18 images is as follows: a set of 0-s exposure shutter images and a set of *T*-s exposure shutter images before Venus shots; a set of 0-s Venus images and a set of *T*-s Venus images; and a set of 0-s shutter images and a set of *T*-s shutter images after Venus shots. The Venus and shutter images with the same exposure time are used to remove noise. The image with 0-s exposure is subtracted from the image with the chosen exposure time; this procedure is called desmearing. As a result of data processing, the signal-to-noise ratio of the UVI image improves to over 100. The above onboard processing is done in the portion of the orbit where Venus moves slowly as seen from *Akatsuki*. The Akatsuki orbit is around the equatorial plane similar to the one of Pioneer Venus (Nakamura et al. 2016). UVI can provide a symmetric view of both hemispheres but no polar view of the planet.

When the spacecraft is close to the planet and moving fast in the percenter, the method of smear correction cannot be performed effectively. In this case, the count in an optical black area of CCD, which is permanently masked outside the imaging area, is used to make the smear correction on the ground.

## Calibration results

### Performance measured before the launch

**Fig. 3:**
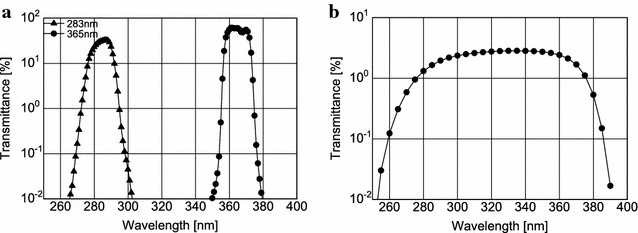
**a** Transmittance of the interference filters for the 365- and 283-nm channels (circles and triangles) and **b** transmittance of diffuser

The transmittances of two interference filters and one diffuser installed in the filter wheel were measured before the launch (Fig. 3a, b). The measurement errors are within the size of symbols in the figures. Both filters have the effective bandwidth of 14 nm, and the transmittance of ~ 60% for 365 nm and ~ 30% for 283 nm. The diffuser has a broad bandpass of 90 nm spanning these two observational wavelengths with the transmittance of a few percent.

The CCD detector adopted for UVI has a UV coating to enhance its quantum efficiency by up to 70% in the observational wavelength range and has a saturation level of 120,000 electrons per pixel. It is one of the full-frame transfer types and has three areas: the image area, storage area, and optical black area. The digital counts of the first two areas are used for data processing at DE, and the counts of the last area are used for smear correction at the ground data processing.

**Fig. 4:**
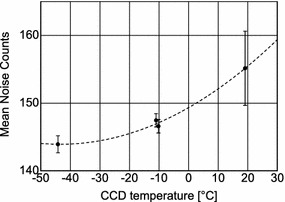
Temperature dependence of the CCD dark counts through the readout electronics

The noise count of the CCD detector through the readout electronics tends to increase rapidly with temperature at the CCD temperature over − 10 °C and is less than 150 counts at a temperature below a few degrees (Fig. 4), achieved by cooling with the radiator under the normal observation mode. The nominal exposure time for the Venus observation is determined as the time where the signal level becomes half the saturation level of the CCD device. The signal-to-noise ratio is over 10 for the raw image before noise reduction and over 100 after the onboard smear correction at the normal observation.

**Fig. 5:**
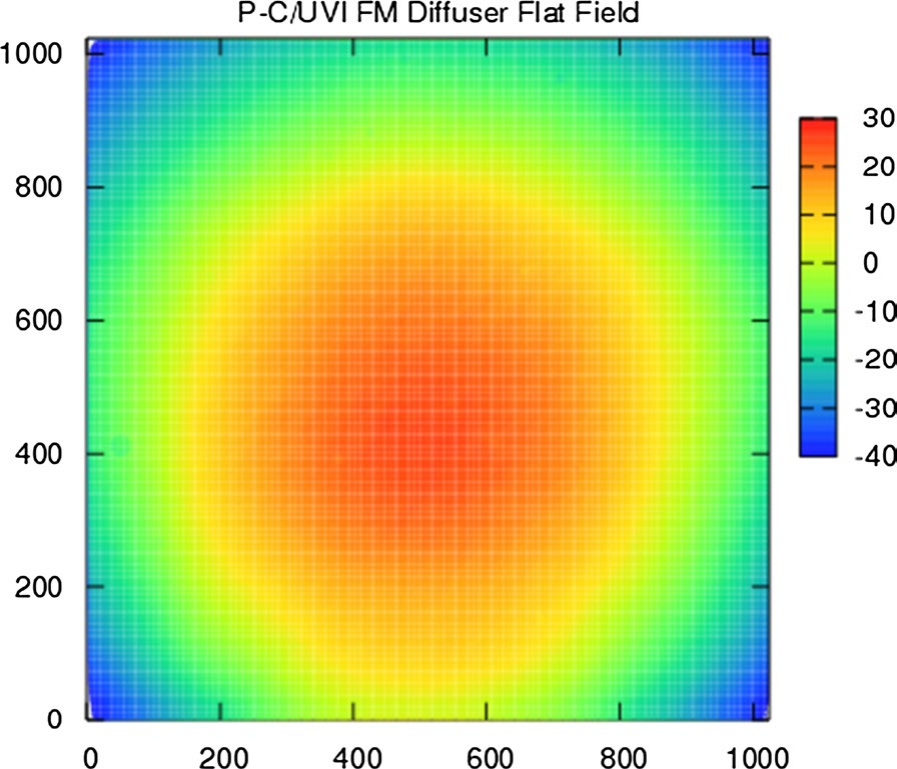
Raw flat image of the integrating sphere using the diffuser with no correction

The flat field image, the distortion image, and the spatial frequency response (SFR) were used to evaluate the total optical performance of UVI-S. The image of a surface light source at the wavelength of 365 nm was obtained using the integrating sphere at the optical facility in the Earth Observation Research Center (EORC) at JAXA. The system is calibrated with the nonuniformity of the area light source of less than 1%. The raw image without any optical correction, such as the cosine fourth law correction due to vignetting, is shown in Fig. 5. The count rate derives the sensitivity of UVI throughout the filter, optical lens, and the CCD detector before the launch. Figure 5 shows that the angular diameter of the integrating sphere aperture viewed from UVI is a 12° circle and that the FOV of UVI is a 12° square. The count rate directed to the center area of the integrating sphere aperture correctly derives the UVI’s total sensitivity. Therefore, the direction of the UVI field-of-view was changed to 3 × 3 directions to obtain the images of the integrating sphere, and the sensitivity of nine areas of the UVI field-of-view was independently calibrated. The averaged image created from the nine images derived the sensitivity of the whole field-of-view.

The flat field pattern was also obtained as Venus images taken by using the diffuser in the orbit around Venus. The diffuser images of Venus are used to create the calibration flat pattern for the data processing on the ground. The UVI image data released from DARTS (Data Archives and Transmission System) of ISAS are corrected by the flat pattern and did not have the clear nonuniformity caused by the sensitivity difference of the CCD detector pixel by pixel. However, the flat pattern is not a perfect correction for photometry analysis that requires careful treatment of the brightness because the brightness gradient of one image remains due to vignetting. Therefore, a factor (a flat conversion factor), based on the pixel sensitivity estimated from the calibration results using the integrating sphere before the launch, is also prepared at DARTS. It is recommended that the released image dataset is mainly used for morphology analysis and that the flat conversion factors are used for photometry studies. The product of the conversion factors and the released data for morphology analysis serves as the absolutely calibrated brightness for photometry analysis.

**Fig. 6:**
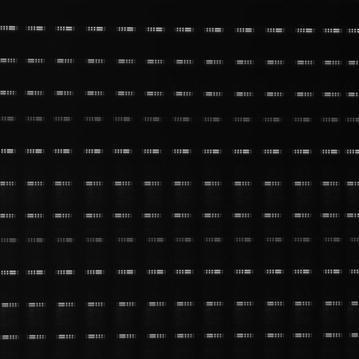
Measured distortion pattern of UVI

The distortion was measured by using black-light lamps with two different sizes of rectangle masks. One pattern has three 3.6-cm and one 10-cm wide masks. Four patterns are set up at one time at a 20.2-m distance from UVI-S with a 36-cm interval. UVI-S is mounted on a tilt-and-swivel base to change its line-of-sight direction to 39 positions (3 elevation angles × 13 azimuth angles). Thirty-nine images are superimposed to create one image shown in Fig. 6. The image reveals that the distortion is − 0.3% at the direction of 5.7° away from the optical axis.

**Fig. 7:**
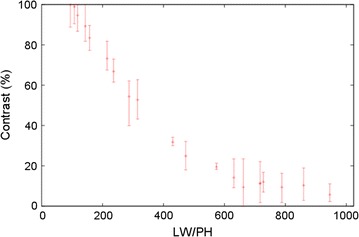
SFR of the UVI imager

Several images of a test chart were taken to estimate the index of the optical performance, SFR (Fig. 7). SFR is measured as a function of the chart pattern frequency of the line width per picture height (LW/PH) and indicates that the limiting resolution of UVI is 650 LW/PH with an SFR of 5%.

### Variation of total sensitivity estimated from the onboard calibration

**Fig. 8:**
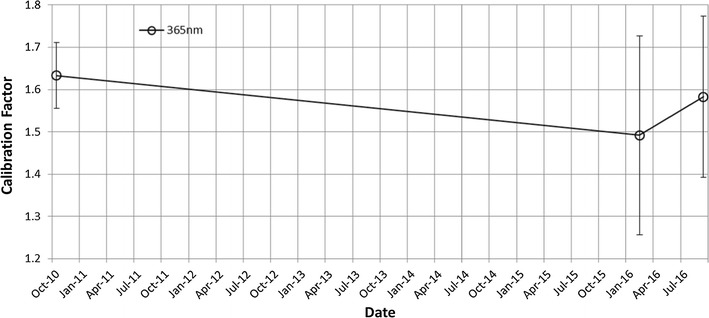
Time variation of calibration factor for the 365-nm channel

The total sensitivity of UVI is obtained from the results of the ground experiments before the launch. The values of the 283- and 365-nm channels, which convert pixel count rate to radiance, are 9227 and 5020 [W/m^2^/sr/m/count rate], respectively. The onboard calibration is performed based on the star observations after the launch. Star fields of Sagittarius and Scorpius were measured during cruising (Oct. 2010; before Venus orbit insertion) and in orbit (Feb. and Sep. 2016). The observed star flux is compared to a known value, so a calibration factor (*β*) can be derived as

$$ \beta = \frac{{F_{\exp } }}{{F_{\text{obs}} }}, $$

where *F*_obs_ is the observed star flux by UVI and *F*_exp_ is the known star flux. *F*_exp_ is calculated as

$$ F_{\exp } = \frac{{\int {T(\lambda )F_{\text{star}} (\lambda )d\lambda } }}{{\int {T(\lambda )d\lambda } }}, $$

where *λ* is wavelength, *T* is the transmittance profile of the 365 nm filter, and *F*_star_ is a star flux spectrum, taken from Pulkovo Spectrophotometric Catalog (Alekseeva et al. 1996; the data are downloaded from http://cdsarc.u-strasbg.fr). The estimation of the calibration factor for the 365-nm channel is shown in Fig. 8.

The observed radiance (W/m^2^/sr/μm) of stars is converted to flux (W/m^2^/μm) by multiplying it with a solid angle of one pixel, *Ω*_pix_ = (0.00021)^2^ square radian. The star flux *F*_obs_ is calculated using the aperture photometry technique, widely used in star flux calculations for ground-based observations (Mighell 1999; Laher et al. 2012). The 283-nm channel is also measured by the same method using data from the International Ultraviolet Explorer (IUE). Table 2 shows the summary of the measured calibration factor β for both of 365- and 283-nm channels.

**Table 2:** Calibration factor β at 365 and 283 nm (avg.)

Date	8 Oct. 2010	8 Feb. 2016	8-9 Sep. 2016
$\bar{\beta }$ (365 nm)	1.63 ± 0.078	1.49 ± 0.24	1.58 ± 0.19
$\bar{\beta }$ (283 nm)	–	–	1.94 ± 0.16

## Initial results

### Operation in the Venus orbit

UVI is activated by executing the observation programs on the DE equipment, and is nominally performed every 2 h; higher observation frequencies are inhibited to maintain the thermal condition of the instrument. UVI images taken so far cover scattering phase angles from 0° to 130°. The scattering property as a function of the phase angle is reported by Lee et al. (2017). The observation programs enable us to perform collaborative observations with the Longwave Infrared Camera (LIR) (Taguchi et al. 2007; Fukuhara et al. 2011, 2017), the 1 μm camera (IR1) (Iwagami et al. 2011, 2018) and the 2 μm camera (IR2) (Satoh et al. 2016, 2017). Some special observations of two UVI shots with short time interval (minimum of 10 min) can be performed around the periapsis. Quasi-simultaneous observations with the radio occultation experiment (Radio Science) (Imamura et al. 2011, 2017) and the Lightning and Airglow Camera (LAC) (Takahashi et al. 2008) are also performed during radio occultations and eclipses.

### Initial Venus images

**Fig. 9:**
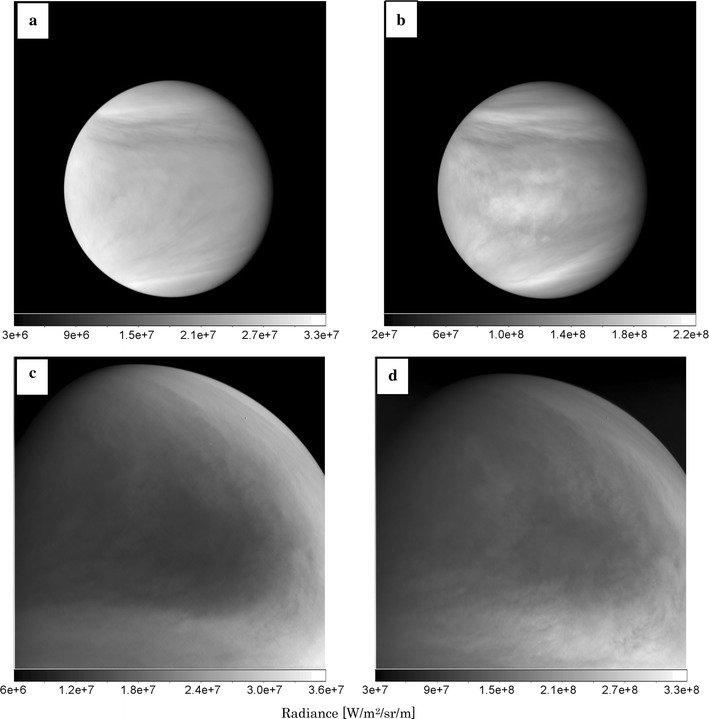
Examples of UVI 283-nm images (**a**, **c**) and 365-nm images (**b**, **d**)

UVI sample images of 283- and 365-nm wavelength are displayed in Fig. 9a, b. The images are taken at 17:14, and 17:17 UTC on April 25, 2016, with exposure times of 0.046 and 0.25 s, at distances of 84,761 and 84,267 km between Venus and *Akatsuki*, respectively. The local time and the latitude at the sub-spacecraft point are 10.5 LT and 2.8°, respectively. The north direction on Venus is at the top of the images. However, the detailed features are quite different from each other. The radiance of a 365-nm image is about ten times larger than that of a 283-nm image. The 365-nm image shows more contrast and bright area in the equatorial region near the center of the image. These differences in UVI images suggest that the spatial distributions of SO_2_ and unknown UV absorbers are governed by, at least partly, different chemical and/or dynamical processes. Another pair of 283- and 365-nm images in low latitude region is shown in Fig. 9c, d, respectively, which are taken at 21:01, and 21:04 UTC on 27 February in 2017. The distance from Venus center is 42,052 and 42,572 km, and the local time and the latitude at the sub-spacecraft are 15.0 LT and − 20.4°, respectively. The significant feature is that there are a large dark area in 283-nm image and a fine cloud complex in 365-nm image. The examples of cloud tracking results are described by Horinouchi et al. (2018), and Limaye et al. (2018) display the simultaneous images with UVI and other cameras.

### Cloud tracking

**Fig. 10:**
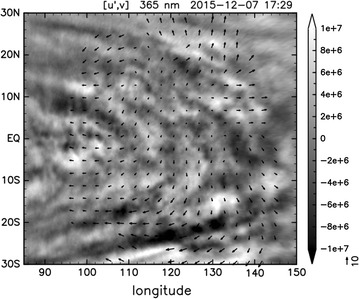
Horizontal velocities derived from three 365-nm images on the day of *Akatsuki*’s Venus orbit insertion (7 December 2015). Arrows show their deviation from the uniform westward wind of 95 m/s. The length scale of the arrows is shown in m/s near the lower-right corner. Grayscale shading shows the high-passed radiance (W/m^2^/sr/m) at the beginning of cloud tracking

UVI images are used to estimate the horizontal winds by tracking cloud features. An example derived from the three 365-nm images taken at 17, 20, 22 h UTC on December 7, 2015, the day of *Akatsuki*’s orbital insertion, is shown in Fig. 10. The tracking is based on the automated method described in Ikegawa and Horinouchi (2016) and Horinouchi et al. (2017a). The method is based on the template matching, but unlike in earlier studies (Kouyama et al. 2012), it utilizes image combinations from more than two images. We utilized a measure of precision based on the sharpness of the cross-correlation surfaces (Ikegawa and Horinouchi 2016), so that the results can be shown in the figures only when the estimated precision is better (smaller) than 10 m/s; a complete description of their derivation is available in the online supplement of Horinouchi et al. (2017b). In equatorial latitudes, the zonal wind exhibits the superrotation at around 100 m/s. The spatial distribution of horizontal winds is consistent with the divergent tidal flow as shown in earlier studies (Del Genio and Rossow 1990); the sub-solar longitude at observation time was 143°; thus, winds vectors in the local afternoon are obtained.

## Summary

Ultraviolet imager (UVI) on *Akatsuki* has successfully started Venus observations. The imager nominally takes a pair of UV images at 283-nm, which is sensitive to SO_2_, and 365-nm, which is in the absorption band of an unknown material, every 2 h by executing the observation programs of the DE application. The spatial resolution is 200 m in the observation from the periapsis of ~ 1000 km altitude, while it is ~ 76 km from the apoapsis of ~ 60 Rv altitude. After the onboard processing, including dark count subtraction, median filtering and desmearing at DE, the signal-to-noise ratio of UVI images reaches over 100, which satisfies the scientific requirement. Correction for the pixel-to-pixel inhomogeneity of the detector sensitivity is made on the ground. The imager has been kept under good temperature conditions during the 5 years of the cruise and does not show any signs of significant degradation.

The images obtained so far show similarities and differences between the two wavelengths. Comparison of the images would provide clues to the three-dimensional distributions of UV absorbers and clouds as well as cloud morphologies. Cloud tracking using sequential images reveals the circulation structure in the cloud top region. The scattering properties at the cloud top altitude in the UV range, such as the phase angle dependence, can be analyzed to reveal the microphysical parameters of cloud particles and constrain the vertical distributions of SO_2_ and unknown absorbers (Lee et al. 2017). UVI has the potential to reveal photochemical and dynamical processes that play crucial roles in the formation of H_2_SO_4_ clouds. UVI data, combined with data from other onboard instruments, are also used for the investigation of the generation mechanism of superrotation.

## Abbreviations

UVI: ultraviolet imager
UV: ultraviolet
SO_2_: sulfur dioxide
CCD: charge-coupled device
H_2_SO_4_: sulfuric acid
H_2_O: water
OCPP: Orbiter Cloud Photo-polarimeter
VMC: Venus Monitoring Camera
UVI-S: sensor of UVI
UVI-AE: analog electronics of UVI
FOV: field-of-view
R_V_: the radius of Venus
DE: Digital Electronics
SFR: spatial frequency response
EORC: Earth Observation Research Center
JAXA: Japan Aerospace Exploration Agency
DARTS: Data Archives and Transmission System
ISAS: Institute of Space and Astronautical Science
LW/PH: line width per picture height
IUE: International Ultraviolet Explorer
LIR: Longwave Infrared Camera
IR1: 1 μm camera
IR2: 2 μm camera
LAC: Lightning and Airglow Camera
UTC: Coordinated Universal Time
LT: local time
